# Inspection of the Defect State Using the Mobility Spectrum Analysis Method

**DOI:** 10.3390/nano12162773

**Published:** 2022-08-12

**Authors:** Il-Ho Ahn, Deuk Young Kim, Woochul Yang

**Affiliations:** 1 Quantum-Functional Semiconductor Research Center, Dongguk University—Seoul, Seoul 04620, Korea; 2 Division of Physics & Semiconductor Science, Dongguk University—Seoul, Seoul 04620, Korea

**Keywords:** deep level transient spectroscopy, mobility spectrum analysis, temperature-dependent minority carrier density, thermally stimulated capacitance

## Abstract

Mobility spectrum analysis (MSA) is a method that enables the carrier density (and mobility) separation of the majority and minority carriers in multicarrier semiconductors, respectively. In this paper, we use the *p*-GaAs layer in order to demonstrate that the MSA can perform unique facilities for the defect analysis by using its resolvable features for the carriers. Using two proven methods, we reveal that the defect state can be anticipated at the characteristic temperature $T_{deep},$ in which the ratio ($R_{N_{n}/N_{h}}$) that is associated with the density of the minority carrier $N_{n}$, to the density of the majority carrier $N_{h}$, exceeds 50%. (1) Using a *p*-GaAs Schottky diode in a reverse bias regime, the position of the deep level transient spectroscopy (DLTS) peak is shown directly as the defect signal. (2) Furthermore, by examining the current–voltage–temperature (I–V–T) characteristics in the forward bias regime, this peak position has been indirectly revealed as the generation–recombination center. The DLTS signals are dominant around the $T_{deep}$, according to the window rate, and it has been shown that the peak variation range is consistent with the temperature range of the temperature-dependent generation–recombination peak. The $T_{deep}$ is also consistent with the temperature-dependent thermionic emission peak position. By having only $R_{N_{n}/N_{h}}$ through the MSA, it is possible to intuitively determine the existence and the peak position of the DLTS signal, and the majority carrier’s density enables a more accurate extraction of the deep trap density in the DLTS analysis.

## 1. Introduction

It is well-known that the effect of defects on electrical properties generates intended or unintended properties, directly or indirectly, on semiconductor devices as well as on materials ranging from catalysis to thermoelectricity. Moreover, defect engineering is one of the hottest topics in material science [1,2,3]. Particularly, the defects in semiconductor devices have a decisive effect on electrical transport characteristics. The transport characteristic is dominated by the minority carrier rather than by the majority carrier. However, it is impossible to extract minority carrier density using the conventional Hall measurement, which is a representative method of extracting carrier density. Fortunately, the mobility spectrum analysis (MSA) method was introduced 35 years ago, which can extract the mobilities and concentrations of the individual carrier species in multilayered semiconductors by using magnetic field-dependent conductivity [4]. Since then, the MSA has developed in various versions [5,6,7,8,9,10,11,12,13,14,15,16,17,18,19,20,21,22,23]. However, there are actually very few cases involving the use of MSA [24,25,26,27,28,29,30,31,32,33,34,35,36,37,38,39,40,41,42,43,44,45,46,47]. Our group has previously published the results of our methodological research on the full maximum-entropy mobility spectrum analysis (FME-MSA) [22], and also presented the results of our research [27,28,45] on transport characteristics as an application of the MSA. Nevertheless, this application is still not actively used.

In this study, we intend to demonstrate that MSA can also be used for defect analysis. We performed the MSA by means of a variable-field Hall measurement using MBE-grown *p*-GaAs epilayers for the demonstration. We obtained the temperature-dependent majority and minority carrier density (and mobility) and found that at the specific temperature $T_{deep}$, a peak appeared in which the ratio $R_{N_{n}/N_{h}}$ of the minority carrier density to the majority carrier density exceeded 50%. We prove that this peak is directly related to the defect level in two ways. (1) A deep level transient spectroscopy (DLTS) [48] measurement was carried out as a direct method in order to examine the activation energy $E_{a}$ of the deep level. For this purpose, a *p*-GaAs Schottky diode was fabricated and the DLTS measurement was performed in the reverse bias regime in order to obtain a DLTS signal. Then, using the $N_{h}$ value extracted through the MSA, the trap density $N_{t}$ at several temperatures was calculated. (2) As an indirect method, the temperature-dependent transport mechanism was analyzed by applying the current transport model to the current–voltage–temperature (I–V–T) measurement values in the forward region of the Schottky diode. We were able to confirm that the $T_{deep}$ reports the correct indication of the temperature position of the DLTS peak using the above two evaluations. As evidence of this, (1) the temperature-dependent minority carrier density peak appears near the $T_{deep}$, which is similar to the DLTS signal. (2) $N_{t}$ was dominant near the $T_{deep}$. (3) $E_{a}$ obtained from the DLTS was comparable to the ratio of the attained minority and majority carrier conductivities from the MSA in the vicinity of the $T_{deep}$. (4) The temperature of the temperature-dependent thermionic emission peak, that was obtained from the temperature-dependent I–V fitting, and the $T_{deep}$, are precisely the same. Furthermore, it was found that the temperature variation range of the maximum peak, according to the rate window of the DLTS spectra, was very similar to that of the temperature-dependent generation–recombination.

These results suggest that the DLTS-like spectra can be obtained using the temperature-dependent minority carrier density ratio by MSA as a unique deep level inspection tool. Furthermore, in the DLTS research, the MSA can serve to determine the exact defect density of the deep level by providing an accurate majority carrier density.

## 2. Sample and Experimental Scheme

**Figure 1:**
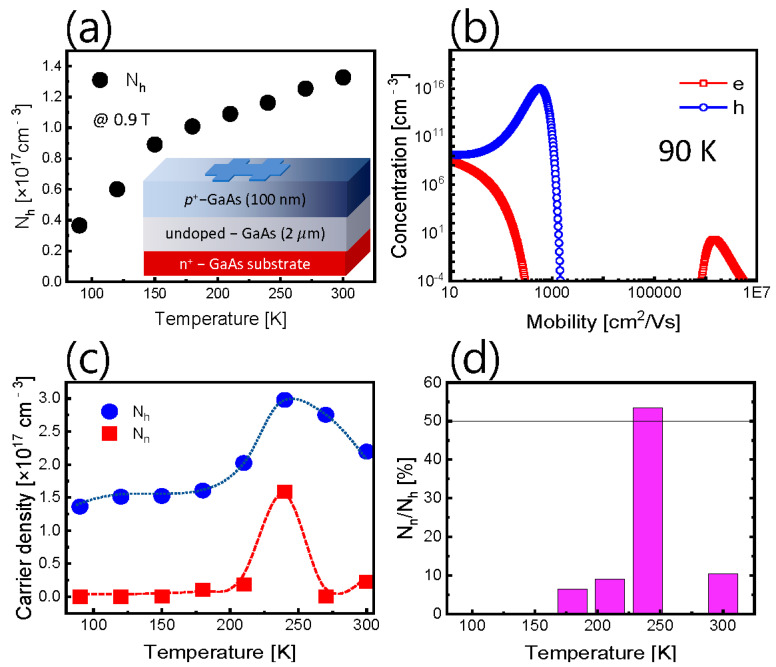
(**a**) Conventional Hall measurement data at 0.9 T. (**b**) Carrier density of the electron and holes at 90 K. (**c**) Temperature-dependent electron and hole density from ME-MSA. (**d**) The ratio of the electron and hole densities from Figure 1c. Note that the temperature position of the maximum ratio above approximately 50% is comparable with the temperature of the DLTS signal found (see Figure 2b,c).

**Figure 2:**
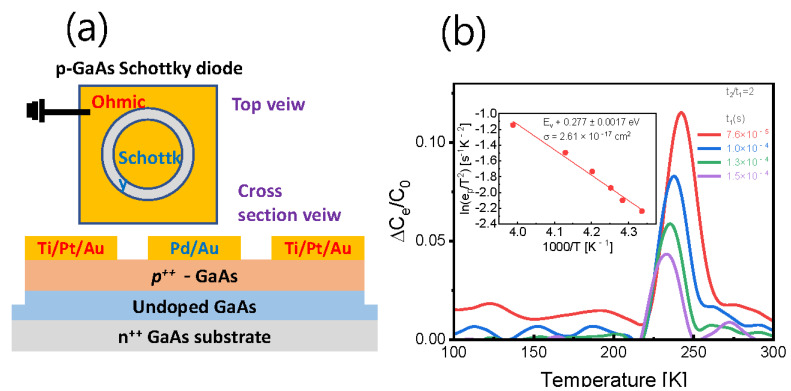

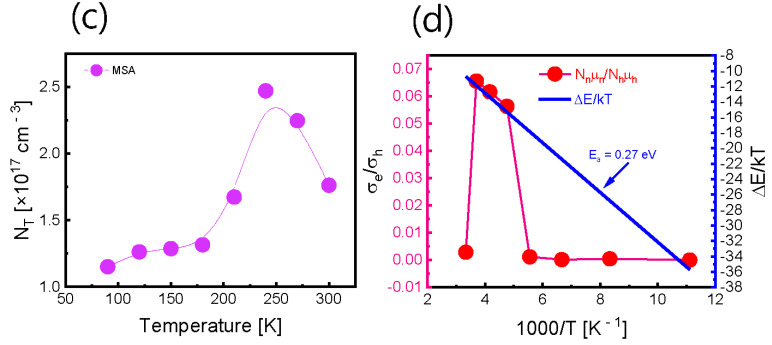
(**a**) Schematic structure of the *p*-GaAs Schottky diode. (**b**) DLTS signals were obtained using the variation in the $t_{1}$ and $t_{2}$ when $t_{2}/t_{1}=2$. The inset shows the associated Arrhenius plot. (**c**) Trap densities calculated from the DLTS method using the majority carrier density obtained from the MSA. (**d**) The slope of the temperature-dependent ratio of the electron and hole conductivity vs. 1/T is similar to $E_{a}/k$ obtained from the DLTS method near the $T_{deep}$.

The inset in Figure 1a schematically illustrates the p-type GaAs epilayer that has been grown onto the (100) GaAs substrate using molecular beam epitaxy (MBE) provided by iSensiRs. Inc., (Jeonju-si, Korea) [49]. Firstly, the GaAs surface was pre-cleaned with thermal heating at 400 °C in the MBE chamber. Then, the 2-μm-thick GaAs buffer layer (<$1\times 10^{16}$ cm^−3^) was grown onto the (100) GaAs surface at 580 °C. Subsequently, the Be-doped GaAs ($2\times 10^{18}$ cm^−3^) layer was grown onto the buffer layer at the same temperature. In order to compare the temperature-dependent carrier concentration, in addition to the conventional Hall measurement, the variable-magnetic-field Hall measurements and the ME-MSA were performed. Ti/Au (20 nm/100 nm) metals were deposited at six edges on the surface of the bar-shaped sample (area = 100 × 520 ${\mu m}^{2}$) for electrical measurements. The Hall coefficient and resistivity were determined in all possible contact configurations for 11 magnetic-field (B) steps in the range from 0 to 1 T. The obtained values were then converted into the conductivity tensor components, which were used for the ME-MSA. The carrier concentrations were calculated from the relationship between conductivity and mobility (see our previous papers [22,28,38,45]). For the I–V and DLTS measurements, a Schottky contact was formed on the top surface of the sample. A round Pd/Au (20 nm/100 nm) Schottky electrode (D~500 μm) was formed onto the *p*-GaAs cap layer. The Ohmic contact metals of Ti/Pt/Au (20 nm/50 nm/100 nm) were also formed onto the *p*-GaAs cap layer in the form of a ring electrode near the circular Schottky contact. (see Figure 2a) Once the Schottky diode structure was fabricated, the I–V and DLTS measurements were performed with a Keithley 2614B and Boonton 7200 system.

## 3. Results and Discussion

### 3.1. Carrier Densities from the Mobility Spectrum Analysis

Figure 1a shows the temperature-dependent majority carrier density obtained from a conventional Hall measurement using a *p*-GaAs Hall bar structure. The conventional Hall measurement method is applicable to a single layer. The simplified structure of the *p*-GaAs sample that we used is shown in the inset of Figure 1a. It is necessary to take into account that, since the Hall coefficient $R_{H}$ ($R_{H}=-\mu /\sigma =-1/qn$) varies with the measured magnetic field B values, the extracted carrier density value appears differently depending on B. The following notations are used: μ is the mobility, σ is the conductivity, q is the elementary charge and n is the carrier density. Figure 1a shows the values of the carrier density using the conventional Hall measurement at 0.9 T. On the other hand, since the MSA is known to provide intrinsic Hall mobility and carrier density, in order to apply this method, the variable-magnetic Hall measurement and ME-MSA were performed. Figure 1b shows the carrier density at 90 K by MSA. The results obtained in the temperature range of 90–300 K are shown in Figure 1c. A clear difference is seen when comparing the results visible in Figure 1a,c. As shown in Figure 1c, the MSA provides the carrier densities for the majority and the minority carriers, respectively. We calculated the ratio $R_{N_{n}/N_{h}}$ relating the carrier density of the minority ($N_{n}$) and majority ($N_{h}$) using the data from Figure 1c and plotted it in Figure 1d. We have found that the relative occupancy of the minority carriers to the majority carrier exceeds 50% near the specified temperature of 250 K. We will now call this characteristic temperature point $T_{deep}$ and discuss the deep level that exists in the vicinity of the $T_{deep}$, using the DLTS method directly and the I–V curve-fitting method indirectly.

### 3.2. Temperature Position of the DLTS Peak

**Figure 3:**
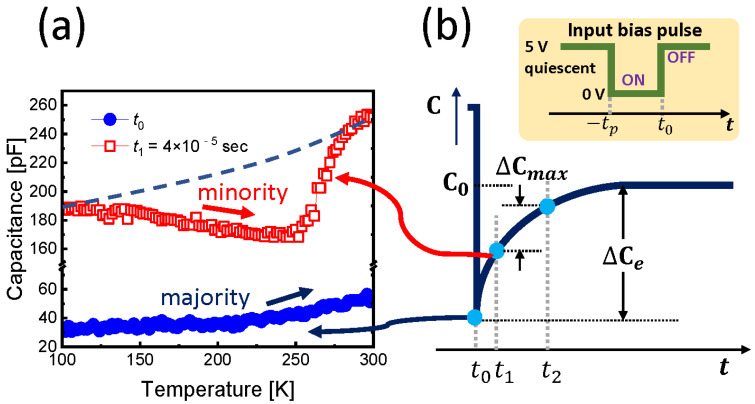
(**a**) TSCAP data in the time $t_{0}$ and $t_{1}=4\times 10^{-5}$ s. The dashed guideline indicates the expected capacitance in the case of the majority carrier. Capacitance decrease with the temperature in the case of $t_{1}$ means that the minority carrier generation is dominant, which correctly coincides with the case of the majority carrier emission (i.e., hole emission) at the deep trap level. Note that here a TSCAP-like measurement was considered at the specific time of the capacitance transient process after the pulse edge time $t_{0}\;$ at (**b**). $t_{1}$, and $t_{2}$ are within 10^−4^ s at the filling pulse time $t_{p}$ = 50 m sec in our test.

**Figure 4:**
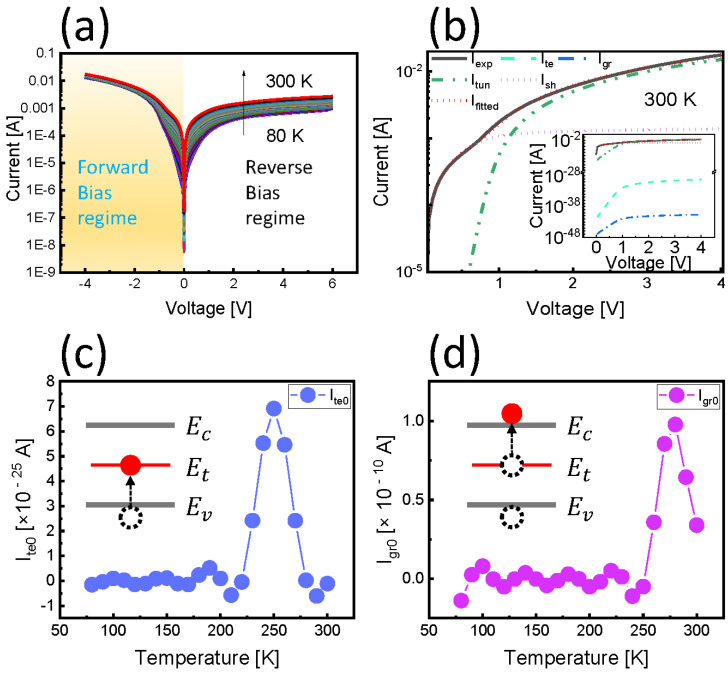
(**a**) I–V–T plot of the Schottky diode from 80 to 300 K. (**b**) I–V fitting with various transport mechanisms. (**c**) The temperature of the maximum peak of the temperature-dependent thermionic emission coefficient also coincides well with that of the DLTS signal found. (**d**) Note that after 250 K, deep traps act as generation–recombination centers. Inset in (**c**,**d**) indicates the generation process when Temperature elevates.

The conventional capacitance-DLTS method was used as the first method to prove that the $T_{deep}$ is correlated with the temperature at which the deep level appears. Figure 2a shows schematically the structure of the *p*-GaAs Schottky diode. The filling bias pulse conditions are shown in Figure 3b, which is carried out in the reverse bias regime of the I–V Schottky diode characteristics, as shown in Figure 4a. Figure 2b shows a plot of the conventional DLTS signal for several time windows defined as $\tau_{m}=\left(t_{1}-t_{2}\right)/ln\left(t_{2}/t_{1}\right)$ when $t_{2}/t_{1}$ = 2. One distinct DLTS peak at a given $\tau_{m}$ is slightly shifted near the 250 K as the $\tau_{m}$ changes.

We will confirm our argument using only this one DLTS peak. Inset in Figure 2d shows an Arrhenius plot of the DLTS signal. The extracted activation energy $E_{a}$ value and the capture cross-section $\sigma_{cap}$ are approximately 0.277 ± 0.0017 eV and 2.61 × 10^−17^ cm^2^, respectively. This result is similar to the previously reported DLTS results for Be-doped GaAs [50].

Figure 2c shows the temperature-dependent trap densities $N_{t}\;$($N_{t}=2N_{a}\frac{\Delta C_{e}}{C_{0}}$) obtained by applying $N_{h}$ data from Figure 1c. (see $\Delta C_{e}$ and $C_{0}$ in Figure 3b). It can be seen that $N_{t}$ increases near the characteristic temperature $T_{deep}$ = 250 K, which proves the proportional dependence of $N_{t}$ on $N_{h}$, and thus shows a similar tendency as in the graph in Figure 1c. Therefore, if $N_{t}$ is obtained using the traditional Hall measurement method, a trend similar to Figure 1a will be observed (data not shown in this paper).

In order to prove whether the trap level can be diagnosed using the mobility and carrier density extracted from the MSA, we employed an assumption that conductivity ($\sigma =q(N_{n}\mu_{e}+N_{h}\mu_{h}))$, where $\mu_{e}$ is the electron mobility and $\mu_{h}$ is the hole mobility, is thermally activated and can be described with the expression $\sigma \propto exp\left(-E_{a}/kT\right),$ where k is the Boltzmann constant. Figure 2d shows the temperature-dependent ratio of electron and hole conductivity $N_{n}\mu_{e}/N_{h}\mu_{h}$. Here, we have found that the slope of $N_{n}\mu_{e}/N_{h}\mu_{h}$ vs. 1000/T is comparable to that of 1000 $E_{a}$/k near the $T_{deep}$, when using the $E_{a}$ = 0.27 eV from the DLTS. This means that deep level energy can also be inferred from the MSA.

### 3.3. Effect of the Minority Carrier Density

We investigated the thermally stimulated capacitance (TSCAP) characteristics using the temperature-dependent capacitance transient data in order to qualitatively prove that minority carriers are generated by thermal energy. This is because TSCAP theoretically shows a tendency to decrease the capacitance value with increasing temperature with respect to the minority carrier [51]. In the DLTS measurement process, we selected an arbitrary time $t_{1}$ from the end of the trap-filling pulse, i.e., from $t_{0}$ to the quiescent state. The TSCAP characteristics for $t_{0}$ and $t_{1}$ are shown in Figure 3a. As expected, at *t* < 0 ($\approx t_{0}$), since the state is in a quasi-thermal equilibrium, the emission of the majority carrier dominates with the increasing temperature, and thus the capacitance tends to increase [51]. On the other hand, even if the electron decay time, which is a minority carrier, is shorter than the transit time, it does not disappear at any $t_{1}$ time during the transient process, and therefore it appears as a diminishing phenomenon in TSCAP. Therefore, Figure 3a proves that the minority carrier becomes dominant near the $T_{deep}$.

### 3.4. Temperature-Dependent Current Transport Mechanism

In the next step, we decided to use the temperature-dependent transport mechanism of the Schottky diode as another proven method in order to understand the relationship between the deep level and the temperature-dependent minority carrier density. By using the I–V curve fitting method in the forward regime of the Schottky diode, a characteristic mechanism can be distinguished. The current transport model used is summarized in Table 1. Figure 4a shows the I–V–T characteristics of the Schottky diode measured from 80–300 K as 2 K increments. Figure 4b shows the fitting result using I–V experimental data at 300 K, using the model defined in Table 1. The characteristic parameters were extracted at all temperatures in the same way. Among the extracted temperature-dependent characteristic parameters, we focused on two components, the thermionic emission and the generation–recombination component, activated by thermal energy. This was due to the well-established knowledge that the deep level is activated by thermal energy and that the trap becomes a generation–recombination center. We were able to conclude that both characteristic values appear between 200 and 300 K in Figure 4c,d. Regarding the DLTS peak, we can expect the hole’s thermal emission, which is the majority trap, to be maximum near the median temperature $T_{deep}$ of the DLTS peak. In other words, the electron density, which is the relatively generated minority carrier’s density, also becomes the maximum point. Therefore, the $T_{deep}$ can be considered as a temperature where the electron density generated by thermal emission is about 50%. Meanwhile, the generation–recombination components dominate up to 250–300 K. This temperature range is considered to be the range in which electrons, that have relatively occupied traps due to the hole emission, are moving towards the conduction band. It can be seen that this temperature range also aligns very well with that of the DLTS peak in Figure 2b, appearing at about 230~300 K when the time window changes. In summary, in Figure 4c, the value of the thermionic emission component $I_{te0}$ clearly shows a maximum close to 250 K. This indicates exactly the same as the maximum point of the minority carrier density extracted from the MSA, as shown in Figure 1c,d. Thus, we demonstrated that the temperature-dependent minority carrier density obtained from the MSA is very similar to the DLTS signal using the Schottky diode.

### 3.5. Consistency of the Carrier Density with the Law of Mass Action

**Figure 5:**
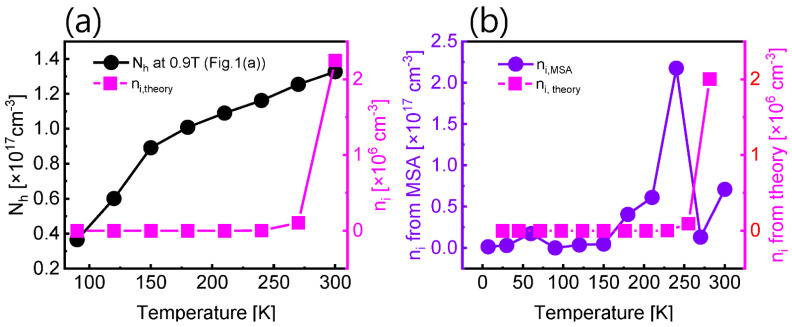
Plot of (**a**) $N_{h}$ versus $n_{i,theory}$ and (**b**) $n_{i,MSA}$ versus $n_{i,theory}$.

In order to verify our methodology, we further checked the temperature-dependence of carrier densities with the law of mass action in semiconductors, which states that the product of the free electron concentration $n_{0}$ in the conduction band and the free hole concentration $p_{0}$ in the valence band is equal to a constant square of intrinsic carrier density at the thermal equilibrium state, i.e., $n_{0}p_{0}={n_{i,theory}}^{2}$. In general, $n_{i,theory}$ is explained as following [53].

(1) $$n_{i,theory}=\sqrt{N_{c}N_{v}}\;e^{-E_{g}/2kT}$$

where $N_{c}\;$($4.7\times 10^{17}$ cm^−3^) and $N_{v}\;$(9$.0\times 10^{18}$ cm^−3^) are the effective density of the state of conduction and the valence band respectively, $E_{g}$ is the band gap energy, k is the Boltzmann constant, and T is the temperature. In Figure 5a, we compared $N_{h}$ observed in Figure 1a with $n_{i,theory}$, in which we assumed $n_{0}\ll p_{0}$ and $p_{0}\approx \;n_{i,theory}$. In general, the temperature dependence of $p_{0}$ in the high-temperature region can be expected to follow the temperature dependence of $n_{i,theory}$ [54]. However, Figure 5a shows that it does not follow the trend well except at 300 K. We could attribute this phenomenon to being mismatched by latent experimental errors or uncertainty in the theoretical model. On the other hand, in Figure 5b, $n_{i,MSA}$ and $n_{i,theory}$ were compared using Figure 1c. Here, we used $n_{i,MSA}=\sqrt{N_{h}N_{n}}$. When compared with Figure 5a, the extracted $n_{i,MSA}$ follows well the temperature dependency of $n_{i,theory}$ in all temperature ranges. However, it does not show a perfect agreement. We think the reason is that the $n_{i,theory}$ itself uses the density of states and the Fermi–Dirac distribution function in intrinsic semiconductors, which are derived in a statistical way. Therefore, we suggest that better theoretical models should be developed in order to describe the semiconductor materials that satisfy the law of mass action. Moreover, we expect that our MSA methodology is employed to study the defect analysis of various materials for obtaining significant information.

## 4. Conclusions

By demonstrating that the temperature-dependent carrier density ratio $R_{N_{n}/N_{h}}$ is related to the deep level, we showed that the MSA can be used to study defects. For demonstration purposes, the temperature-dependent variable-field Hall measurements and the MSA were performed using the *p*-GaAs layer in order to obtain the majority and minority carrier densities in the temperature range of 90–300 K. We found that near the specific temperature $T_{deep}$, the ratio $R_{N_{n}/N_{h}}$ of the minority carrier density $N_{n}$ to the majority carrier density $N_{h}$ exceed 50%. The correlation of this characteristic temperature $T_{deep}$ with the deep level was explained by the performed DLTS and I–V fitting analyses using the Schottky diode. Near the $T_{deep}$, the trap activation energy from the DLTS was comparable to the ratio of the attained minority and majority carrier conductivities from the MSA. The $T_{deep}$ coincided exactly with the temperature of the temperature-dependent thermionic emission peak. In addition, we found that the temperature range of the DLTS peaks change when the time window variation was consistent with the temperature-dependent generation–recombination peak range around the $T_{deep}$. We have therefore demonstrated that this characteristic temperature $T_{deep}$ can serve as a very good indicator in order to suggest the existence of a deep level. Our findings have the following implications. (1) The existence of a deep level can be intuitively judged only by the MSA without the DLTS analysis. (2) More importantly, it provides an exact majority carrier density value for the accurate $N_{t}$ calculation in the DLTS analysis. We expect defect testing through MSA to be conducted more actively.

## Figures and Tables

**Table 1:** Physical mechanisms of the electrical conduction of the *p*-GaAs Schottky diode. We obtained the current–transport components by adopting all of the detailed fitting methods in Ref. [52].

Transport Mechanism	Current Model	
	Characteristic Component	I–V Model
Thermionic emission	$I_{te0}$	$I_{te}=I_{te0}\left[exp\left\{\frac{q(V-IR_{s}}{k_{B}T}\right\}-1\right]$
Generation–recombination	$I_{gr0}$	$I_{gr}=I_{gr0}\left[exp\left\{\frac{q(V-IR_{s}}{2k_{B}T}\right\}-1\right]$
tunnelling	$I_{tun0}$	$I_{tun}=I_{tun0}\left[exp\left\{\frac{q(V-IR_{s}}{E_{0}}\right\}-1\right]$
leakage	-	$I_{lk}=\frac{V-IR_{s}}{R_{t}}$

## Data Availability

The data presented in this study are available on request from the corresponding author.
